# Effects of Amaranth Seed- and Bambara Groundnut-Based Media on the Aroma-Active Volatile and Amino Acid Profiles of Oyster Mushroom (*Pleurotus ostreatus*) Mycelia

**DOI:** 10.3390/foods15091584

**Published:** 2026-05-04

**Authors:** Kayise Hypercia Maseko, Margaux Lim Ah Tock, Alvaro Viljoen, Paul Bartels, Thierry Regnier, Belinda Meiring

**Affiliations:** 1 Department of Biotechnology and Food Technology, Tshwane University of Technology, Private Bag X680, Pretoria 0001, South Africa; masekokayise@gmail.com (K.H.M.); regniert@tut.ac.za (T.R.); 2 Department of Pharmaceutical Sciences, Tshwane University of Technology, Private Bag X680, Pretoria 0001, South Africa; limahtockmj@tut.ac.za (M.L.A.T.); viljoenam@tut.ac.za (A.V.); 3 WildBio Company, Pretoria 0001, South Africa

**Keywords:** *Pleurotus ostreatus*, oyster mushroom, mycelial fermentation, aroma-active volatile compounds, HS-SPME GC–MS, amino acid profile, plant-based media, amaranth seed, Bambara groundnut, functional food ingredients

## Abstract

The growing demand for sustainable alternative proteins has intensified interest in fungal mycelia as a nutrient-dense biomass for food applications. This study compared *Pleurotus ostreatus* fruiting bodies with mycelia grown in liquid state on amaranth seed- and Bambara groundnut-based media, evaluating aroma-active volatiles and amino acid composition. Across 52 identified volatiles, C8 oxylipin-derived compounds dominated all matrices, with exceptionally high odour activity values (OAVs) for 1-octen-3-one (~4.1 × 10^3^), 3-octanone (~1.5 × 10^3^), 1-octen-3-ol (~8.3 × 10^2^) and 3-octanol (~5.3 × 10^2^). Amaranth-grown mycelia showed intensified mushroom/green/fatty notes due to elevated C8 ketones and unsaturated aldehydes, whereas Bambara-grown mycelia exhibited reduced C8 prominence and stronger malty, nutty and fermented nuances driven by Ehrlich-pathway aldehydes (e.g., 3-methylbutanal ~2.0 × 10^3^), with floral contributions from linalool (~3.8 × 10^2^). Mycelial protein contents ranged from 35.8 to 36.1 g/100 g (amaranth) and up to 38.2 g/100 g (Bambara), compared with 39.5 g/100 g in the fruiting body. Amino acid scores (AAS) identified cystine + methionine as limiting; mycelia exhibited higher AAS, with more indispensable amino acids exceeding reference requirements. Elevated glutamic and aspartic acids underscore the umami potential of the mycelial biomass. Overall, these plant-based substrates can strategically modulate both flavour chemistry and amino acid balance in *P. ostreatus* mycelia, supporting their use as nutritionally relevant, flavour-active ingredients in alternative protein and hybrid food systems.

## 1. Introduction

Global food systems are under increasing pressure from population growth, climate-related constraints and the high environmental cost associated with animal-based protein production [1,2]. These challenges have accelerated the search for sustainable alternative proteins, including plant-, fungal- and microbe-derived options [3]. Within this landscape, fungal mycelia are increasingly recognised as nutrient-dense, sustainable protein sources with favourable amino acid profiles and applicability in structured food matrices, such as hybrid meat analogue products [4,5]. Compared with fruiting bodies, mycelia can be cultivated in controlled liquid-state systems with substantially shorter production cycles and reduced spatial requirements, enabling improved scalability and more consistent biomass composition.

*Pleurotus ostreatus* (oyster mushroom) is widely cultivated and valued for its nutritional attributes, texture and the presence of aroma-active volatile compounds responsible for mushroom-like, green, fruity and fatty sensory notes [6]. Its volatilome is characterised by aldehydes, ketones, alcohols and particularly C8 compounds (e.g., 1-octen-3-ol, 1-octen-3-one). Biosynthesis of these compounds involves lipid oxidation pathways, including lipoxygenase-mediated reactions [7], and amino-acid catabolic pathways such as the Ehrlich mechanism, which produces fusel alcohols and aldehydes from branched-chain and aromatic amino acids [8]. The relative abundance of these compounds determines sensory quality and is highly sensitive to growth conditions and substrate composition.

Culture medium composition is a major determinant of fungal metabolic output. Early studies found that volatile profiles differ substantially between *P. ostreatus* mycelia and fruiting bodies across culture modes [9], while more recent work highlights the effects of temperature, pH and nutrient availability on enzymatic activity and metabolite formation [10]. Despite growing interest in liquid-state mycelial cultivation for biomass, enzymes, and specialised metabolites [11], limited information exists on how nutrient-rich, plant-based substrates alter volatile production and amino acid composition in *P. ostreatus* mycelia. Notably, recent work on other basidiomycetes shows marked substrate-dependent shifts in protein quality and flavour-active metabolites, highlighting that similar dynamics may also be expected in *P. ostreatus* but remain uncharacterised [12,13].

Our previous experimental work demonstrated that amaranth seed flour- and Bambara groundnut flour-based media produced mycelia with high yield and protein content under controlled liquid-state cultivation [14]. A summary of the key yield and protein outcomes from this optimisation study is provided in Table 1. This established these plant-based substrates as promising candidates for producing nutrient-enhanced mycelia. However, for applications such as hybrid meat analogue development, protein enrichment alone is not sufficient: flavour, particularly the aroma-active volatile composition, and amino acid balance are also critical determinants of ingredient quality and consumer acceptance. Therefore, understanding how nutrient sources modulate sensory-relevant metabolites is essential for selecting mycelia with optimal characteristics for functional food applications.

Amaranth (*Amaranthus* spp.) and Bambara groundnut (*Vigna subterranea*) flours were selected as media due to their nutrient density and relevance as underutilised African crops. A comparative summary of their reported nutritional composition is provided in Table 2.

Amaranth seed flour is characterised by relatively high protein content, a lysine-rich amino acid profile, and lipid fractions dominated by linoleic and oleic acids, bioactive components such as squalene, tocopherols, and phenolic compounds [15,16,19]. Bambara groundnut flour generally provides comparable or higher protein levels, with a legume-typical amino acid profile and lipid fractions rich in unsaturated fatty acids and palmitic acid, as well as bioactive components such as polyphenols, flavonoids, tannins, phytic acid and oxalate [20,21,22,23]. These nutritional and bioactive profiles suggest that the two substrates may differentially influence fungal amino acid biosynthesis and aroma-relevant metabolic pathways, yet their specific impacts on *P. ostreatus* mycelial amino acid and volatile profiles have not previously been investigated.

The objective of this study was to investigate the effects of amaranth seed flour- and Bambara groundnut flour-based liquid media on the aroma-active volatile profiles and amino acid composition of *P. ostreatus* (HK35) mycelia across different culture durations, and to compare these with the profiles obtained from the fruiting bodies. By integrating volatile profiling with amino acid analysis under controlled liquid-state conditions, this work addresses a key knowledge gap and supports the development of flavour-optimised, protein-enhanced mycelial ingredients for functional foods and hybrid meat analogue product development.

## 2. Materials and Methods

### 2.1. Materials

Culture media for the cultivation of mycelia composed of amaranth seeds (*Amaranthus*) were purchased locally from Wellness Warehouse (Pretoria, South Africa) and Bambara groundnut (*Vigna subterranean*) was purchased from Republic Fisheries and Meat Market (Pretoria, South Africa). All chemicals and reagents used in this study were of analytical grade. Malt Extract Agar (MEA), internal standard 4-methyl-1-pentanol, and C_8_–C_40_ alkane calibration standard for the calculation of linear retention indices (RIs) were purchased from Sigma-Aldrich (Johannesburg, South Africa).

### 2.2. Fungi Sample Preparation

Freshly harvested fruiting bodies of *P*. *ostreatus* (HK35 strain), cultivated on a wheat straw substrate supplemented with 15% wheat bran, 2% calcium carbonate, and 5% coffee residue (composited for 30 days), were sourced from Mzansi Mushrooms (Johannesburg, South Africa), and used as the control sample. The same *P*. *ostreatus* strain utilised for mycelial biomass cultivation was obtained from Sylvan Africa (Pty) Ltd. (Centurion, South Africa). The strain was maintained at 4 ± 1 °C and sub-cultured by inoculating mycelial plugs onto MEA plates, which were incubated at 25 ± 1 °C for 8 days, as described previously [14].

Mycelia biomass was produced using liquid-state culture with two different nutrient media consisting of either amaranth seed flour or Bambara groundnut flour. Cultivation was conducted in 250 mL Erlenmeyer flasks under identical static surface culture conditions. Each culture medium was prepared by mixing 4 g of the respective nutrient flour and adjusting to 50 mL with distilled water [14]. The flasks were plugged with cotton wool, covered with aluminium foil, and sterilised in an autoclave at 121 °C for 15 min. After sterilisation and cooling under sterile laminar airflow, 4 units of 6 mm mycelial plugs (active growth state) were inoculated onto the centre of each medium. The cultures were incubated at 25 ± 1 °C for periods of 8, 10, 12, and 14 days before harvesting the biomass.

The mycelial mat was aseptically collected from the culture flask to ensure the integrity of the biomass. Thereafter, the mat was carefully washed with sterile distilled water to remove residual medium components and potential contaminants. The cleaned biomass was then transferred into a sterile container to maintain asepsis and was freshly analysed.

### 2.3. Headspace Solid-Phase Microextraction Procedure

Immediately after harvest, samples of fresh mushroom fruiting bodies and mycelia biomass (representing distinct cultivation periods and nutrient medium type) were processed as previously described [6]. Each sample was finely chopped and homogenised before weighing 1.00 ± 0.01 g into a 20 mL glass headspace vial fitted with a screw cap and Teflon-coated septa (Supelco Inc., Bellefonte, PA, USA). Subsequently, 10 µL of an internal standard solution (4-methyl-1-pentanol, 1 mg/mL in methanol) was added. To facilitate volatile equilibration and inhibit sample deterioration, 4 mL of a saturated aqueous sodium chloride (NaCl) solution (20% *w*/*v*) was pipetted into the vial and gently mixed [6].

Analysis was conducted in triplicate employing automated headspace solid-phase microextraction (HS-SPME) using a Gerstel MPS Autosampler (Leco Africa (Pty) Ltd., Kempton Park, South Africa). Volatile compounds were extracted by adsorption using a 65/10 µm polydimethylsiloxane/divinylbenzene-overcoated (PDMS/DVB-OC) StableFlex stainless steel fibre (Supelco Inc., USA). This fibre was selected for its high affinity toward volatile polar compounds such as low-molecular-weight aldehydes, alcohols, and ketones, as well as a broad range of other volatile analytes [24,25,26]. The adsorption process is known for its efficiency and rapid release [25]. Before analysis and between runs, the fibre was conditioned at 200 °C for 30 min.

The samples were equilibrated for 5 min prior to fibre insertion and maintained at 50 °C throughout the 40 min extraction. After adsorption of the volatiles from the headspace, the coated fibre was retracted from the headspace and inserted into the gas chromatograph (GC) inlet, where desorption of volatile compounds occurred for 0.5 min at 250 °C for subsequent analysis.

### 2.4. Gas Chromatography Analysis

#### 2.4.1. Time-of-Flight Mass Spectrometry (ToF-MS)

Chromatographic analysis of the headspace volatiles was performed using a Leco Pegasus 4D GC system (Leco Africa (Pty) Ltd.), comprising an Agilent 6890A GC (Agilent Technologies, Waldbronn, Germany) and a Gerstel MPS autosampler (Gerstel, Mulhheim, Germany). A specialised SPME glass inlet liner (Restek, Bellefonte, PA, USA) was employed, and the analysis was conducted in splitless mode.

Separation of compounds was achieved using a Stabilwax capillary column (cross-bond polyethylene glycol, Restek, Bellefonte, PA, USA) measuring 30 m in length, 0.25 mm internal diameter, and 0.25 μm film thickness. Helium (Afrox, South Africa) served as the carrier gas at a constant flow rate of 1.00 mL/min. The primary column oven was programmed with an initial temperature of 35 °C (held for 2 min), followed by a ramp of 20 °C/min to 100 °C, then a gradual increase of 5 °C/min up to 240 °C. The GC analysis time was 33.25 min. The MS transfer line was maintained at 250 °C, with the ion source maintained at 200 °C. An ionisation energy of 70 eV was applied and data were acquired at 10 spectra per second over a mass-to-charge ratio (*m*/*z*) range of 35–550.

#### 2.4.2. Identification and Semi-Quantification of Volatile Compounds

Compounds were tentatively identified by matching their relative retention indices (RRIs), calculated using a C_8_–C_40_ alkane standard mixture (Sigma-Aldrich, Johannesburg, South Africa), with reference values reported in the National Institute of Standards and Technology (NIST) Webbook [27] and other literature sources. An acceptance criterion of ±30 units was applied for RRI matching. Additionally, mass spectra were compared against the NIST11 library, with matches exhibiting a similarity score of 70% or higher considered reliable. During data processing, peaks were evaluated based on peak width, and a signal-to-noise (S/N) ratio greater than 100 was considered. Data processing and instrument control were performed using Leco^®^ ChromaToF-GC Software Version 4.51.6.0. Semi-quantitative analysis was conducted by calculating peak areas relative to the internal standard, which was added at a concentration equivalent to 10.0 µg per g of sample. The “Good Scents Company Information System” database was used for the characterisation and description of volatiles [28].

### 2.5. Protein and Amino Acid Analysis

Freshly harvested fruiting bodies and mycelia samples were frozen at −80 °C for at least 48 h. The frozen samples were lyophilised using a vacuum freeze dryer for approximately 5 days, ground into powder using a mortar and pestle, vacuum-sealed and stored at −80 °C until further analysis [29].

Crude protein content of the lyophilised samples was determined according to the Association of Official Agricultural Chemists (AOAC) method 960.52, employing the Dumas combustion technique. Analysis was performed using a Leco TruMac^®^ N analyser (Leco Corporation, St. Joseph, MI, USA). All measurements were conducted in triplicate, and crude protein percentage was estimated by multiplying the nitrogen content by a conversion factor of 5.70.

Amino acid content quantification of both mushroom and mycelia samples was carried out at the Food and Drug Assurance (FDA) Laboratories^®^ (Pretoria, South Africa), using liquid chromatography–tandem mass spectrometry (LC-MS/MS) (AB Sciex 4000QTRAP, Concord, ON, Canada). Protein hydrolysis was performed using acid and alkaline digestion methods. Most amino acids were released through acid hydrolysis with 6 M hydrochloric acid (HCl), while tryptophan was specifically quantified following alkaline hydrolysis using 4 M sodium hydroxide (NaOH). Hydrolysis was conducted at 110 °C for a minimum of 25 h in sealed, heat-resistant culture tubes to ensure complete protein digestion.

Cystine and methionine contents were determined following performic acid oxidation, whereas tryptophan content was assessed separately through alkaline hydrolysis. The hydrolysed amino acids, except for tryptophan, were separated on a Phenomenex Luna C18 column (150 × 2 mm, 5 µm particle size) using a 15 min gradient of ion-paired mobile phases at a flow rate of 0.25 mL/min. Tryptophan separation was performed on the same column but with a 10 min gradient of acidified water and acetonitrile. Amino acid quantification was achieved through LC-MS/MS using electrospray ionisation in positive mode, controlled by Analyst Software to ensure precise results.

The limiting amino acid score (AAS) was calculated to estimate the protein quality of the mycelia and fruiting body by comparing their amino acid composition against adult reference patterns established by the Food and Agriculture Organization and World Health Organization [24,25]. In reference patterns, methionine and cystine, and phenylalanine and tyrosine are combined as metabolically related amino acids. For consistency and due to analytical constraints, the concentrations of leucine and isoleucine were also combined in this study for comparative evaluation.

### 2.6. Statistical Analysis

Analysis was conducted in triplicate, and data were presented as mean ± standard deviation. Differences between means were established by one-way analysis of variance (ANOVA) and Duncan’s multiple-range test with the Statistical Package for Social Sciences (SPSS) programme 29.0 software (IBM SPSS Inc., Chicago, IL, USA). Descriptive statistics were calculated in SPSS, and box plots were used to visualise the distribution of amino acid concentration across amaranth-based and Bambara-based mycelia, and culture periods (days). Given the interconnected dataset of the 17 amino acid measurements, box plots provided a visual representation of the distribution.

## 3. Results and Discussion

### 3.1. Volatile Compounds Detected and Identified

As detailed in Table 3, GC-MS analyses detected and identified 52 volatile compounds across the fresh *P. ostreatus* fruiting body and mycelial samples. The mycelia cultivated on the two nutrient media exhibited clear differences, with 47 compounds identified in mycelia grown on the amaranth seed-based medium and 35 in those grown on the Bambara groundnut-based medium. In contrast, only 17 compounds were detected in the fruiting body, which is considerably fewer than the 36 compounds reported in fresh *P. ostreatus* mushrooms (CS4, CS5, CS6 and LGAM3002 strains) cultivated on wheat straw, wheat-straw–grape marc mixtures, and olive-leaf/olive-mill-waste substrates [6]. These findings confirm that liquid-state culture of mycelia yields a greater diversity of volatile compounds than the fruiting body, aligning with previous reports that mycelia have a richer volatile profile in *P. ostreatus* mycelium relative to the fruiting body [9].

Across all samples (*P. ostreatus* fruiting bodies and mycelia cultivated on the two nutrient media), 14 volatile compounds were shared, representing aldehydes, ketones, alcohols, and aromatic compounds that comprise the species’ core volatilome. These included mushroom-associated volatiles such as 3-octanone, 3-octanol, 1-octen-3-ol, 1-octanol, benzaldehyde, toluene, 2-pentylfuran, and (E)-2-octenal. This pattern matches the current understanding of *P. ostreatus* aroma chemistry, where alcohols and ketones (particularly C8 compounds) are described as dominant contributors to mushroom aroma [6,9]. The recurrence of these compounds across all samples suggests a conserved metabolic backbone in *P. ostreatus*, independent of nutrient source or developmental stage. Compounds such as heptanal, octanal, nonanal and linalool were detected in both mycelial matrices but were absent in the fruiting body in this dataset.

The Bambara-grown mycelia produced three unique volatiles, 2-methylbutanal, (Z)-2-octen-1-ol, and acetophenone, that were not detected in either the fruiting body or the amaranth-grown mycelia. Conversely, the amaranth-grown mycelia produced 14 unique compounds: 3-methyl-2-butanone, pentanal, 3-heptanone, 2-octanone, 4-methyl-6-hepten-3-one, 5-methyl-5-hepten-3-one, 6-methyl-5-hepten-2-one, 2-nonanone, 4-ethylcyclohexanone, methyl-1-methylcyclopropyl ketone, 2-ethyl-1-hexanol, 2-undecanone, octanoic acid, and nonanoic acid, that were not detected in the other two matrices. The fruiting body contributed two unique compounds, namely methyl 4-methylpentanoate and methyl tridecanoate.

The compound 4-ethylcyclohexanone, identified exclusively in amaranth seed-based mycelia, was previously detected in fermented shrimp paste [24] and noted as a key contributor to desirable fermentation-derived flavour attributes. Octanoic acid and nonanoic acid, also detected only in amaranth-based mycelia, are medium-chain fatty acids known to be contributors to fungal aroma and microbial flavour development, as demonstrated in *Monascus*-fermented cheese [30]. Their roles in spontaneous wine fermentations have also been highlighted, underscoring their wider metabolic significance [31]. Several ketones unique to amaranth-grown mycelia, including 3-methyl-2-butanone, 3-heptanone, 4-methyl-6-hepten-3-one, and 6-methyl-5-hepten-2-one, are recognised for their pleasant sweet, fruity, or floral notes, contributing to their value as natural flavour components [32].

Across all samples, the dominant odour descriptors were sweet, fruity, green, and fatty. Mushroom-like odours were strongly associated with 3-octanone, 3-octanol, and 1-octen-3-ol in both mycelial media, whereas 1-octen-3-one occurred only in the fruiting body and amaranth-based mycelia, and was absent in the Bambara-based mycelia. The alkanes 2-methyldecane and 3-methyldecane, which lack formal sensory descriptions, were previously identified in cultures of *Fusarium verticillioides* [33] and *Laetiporus sulphureus* [34]. Methyl tridecanoate, one of the few compounds unique to the fruiting body, has also been reported in the fruiting bodies of *Agaricus bisporus* (button mushroom), *Pleurotus ostreatus* (pearl oyster mushroom) and *Lentinula edodes* (shiitake mushroom) [35], confirming its broader distribution among edible mushrooms.

Although volatile profiles can vary widely among fungal genera, species and even individual isolates [6], the compounds detected exclusively in the Bambara groundnut–based mycelia are not anomalous; rather, they reflect well-established biochemical and sensory functions in fungi. Acetophenone, for example, is a natural aromatic ketone produced by various fungi and plants and is widely used in fragrance formulations due to its sweet, pungent and floral odour profile [36], while several acetophenone derivatives contribute orange-blossom-type notes in food applications [37]. The aldehyde 2-methylbutanal, also unique to Bambara-based mycelia, has previously been identified in *Fusarium graminearum* cultivated on yeast-extract sucrose agar [38]. This compound imparts malty, nutty and fermented aromas, and together with 3-methylbutanal, forms part of a key group of Strecker aldehydes associated with Maillard-type reactions. These aldehydes are known contributors to roasted-meat flavour notes, such as roasted lamb [39]. Collectively, the presence of such compounds underscores the biochemical versatility of *P. ostreatus* mycelia and highlights how nutrient source can direct the formation of aroma-active metabolites with distinct sensory relevance.

### 3.2. Semi-Quantification of Volatile Compounds

**Figure 1:**
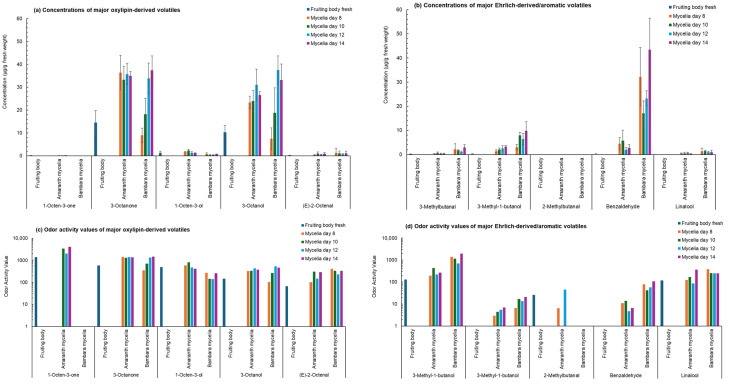
Key aroma-active volatile compounds identified in *Pleurotus ostreatus* fruiting bodies and mycelia cultivated on amaranth seed- and Bambara groundnut-based media. Panels (**a**,**b**) show concentrations (µg/g fresh weight, mean ± SD, *n* = 3) of selected oxylipin-derived (**a**) and amino acid-derived/aromatic (**b**) volatiles, while panels (**c**,**d**) present the corresponding odour activity values (OAVs) for the same compounds. Compounds were selected based on high concentrations and/or high OAVs, representing the major contributors to aroma differentiation between substrates. Full quantitative data for all detected volatiles are provided in Appendix A.

Fresh fruiting body samples and mycelia cultivated on amaranth seed-based and Bambara groundnut-based media across four culture periods produced a diverse range of volatile compounds, as shown in Appendix A, with key aroma-active compounds summarised in Figure 1. Across all samples, 14 alcohols (0.02–37.5 µg/g), 14 ketones (0.02–37.4 µg/g), 11 aldehydes (0.01–43.4 µg/g), 3 esters (0.04–1.22 µg/g), 3 aromatic compounds (0.01–3.23 µg/g), 2 acids (0.11–0.30 µg/g), 2 alkanes (0.01–0.09 µg/g), one benzene derivative (0.15–1.06 µg/g), one terpene (0.32–1.41 µg/g), and one co-eluted entry (1.09–4.63 µg/g) were identified. Compounds reaching the highest concentrations included 3-octanone (up to 37.4 µg/g), 3-octanol (37.5 µg/g), benzaldehyde (43.4 µg/g), 1-hexanol (7.77 µg/g), and 3-methyl-1-butanol (9.81 µg/g).

To identify the volatiles most likely to influence aroma, odour activity values (OAVs) were computed as the ratio of the measured concentration to the water odour threshold; by convention, OAV > 1 suggests a potential perceptible contribution [8]. Water-based odour threshold values were used because water constitutes the dominant fraction of both oyster mushroom fruiting bodies and mycelial biomass produced under liquid-state cultivation, making water the most appropriate comparative matrix currently available. Figure 1 summarises the cumulative odour activity contributions of major volatile compound classes across fruiting bodies and mycelia cultivated on the two plant-based media, revealing clear matrix-level shifts in aroma balance and indicating that a limited number of dominant compounds and metabolic pathways account for most of the observed aroma-relevant chemical signal.

Several analytes presented very high OAVs and are therefore expected to shape the overall flavour profile: 1-octen-3-one (~4.1 × 10^3^), 3-methylbutanal (~2.0 × 10^3^), 3-octanone (~1.5 × 10^3^), 2,4-decadienal (~1.3 × 10^3^), 1-octen-3-ol (~8.3 × 10^2^), 3-octanol (~5.3 × 10^2^), (Z)-2-heptenal (~5.3 × 10^2^), (E)-2-nonenal (~4.9 × 10^2^), (E)-2-octenal (~4.1 × 10^2^), and linalool (~3.8 × 10^2^). The prominence of C8 compounds in both fruiting body and mycelial samples is characteristic of *Pleurotus* species and reflects activity of the lipoxygenase-derived oxylipin pathway [6]. In this mechanism, linoleic acid is released from membrane lipids by phospholipases and oxidised by lipoxygenase to form 10-hydroperoxy-8,12-octadecadienoic acid (10-HPOD). Hydroperoxide lyase then cleaves 10-HPOD to yield 1-octen-3-ol, the primary “fresh mushroom” alcohol, which can be enzymatically oxidised or reduced to 1-octen-3-one, 3-octanone and 3-octanol [8]. This mechanism explains the dominant OAV contributions of these volatiles in the present study.

The fruiting body showed the expected prominence of 1-octen-3-ol, 1-octen-3-one and 3-octanone, all well above threshold, consistent with oxylipin-derived C8 compounds being central to fresh mushroom aroma in *P. ostreatus* [6,8]. By contrast, mycelial cultures, especially under submerged conditions, commonly produce a broader or shifted set of volatiles compared with fruiting bodies [9].

Amaranth seed-based mycelia were characterised by a ketone-rich, C8-intensified profile. The main OAV driver was 3-octanone (~1.5 × 10^3^), while 1-octen-3-one (absent in Bambara-grown mycelia) reached ~4.1 × 10^3^ and contributed strongly to the earthy, mushroom–metallic character. Other ketones, such as 3-heptanone, were near threshold (OAV ~1–2) and thus contributed minimally. Although octanoic and nonanoic acids were detected exclusively in amaranth-grown mycelia, their concentrations were below the threshold (OAV < 1). Overall, the amaranth profile aligns with enhanced oxylipin activity, leading to intensified mushroom, green and fatty nuances derived from C8 alcohol/ketone interconversion [7].

In Bambara groundnut-based mycelia, the OAVs pointed to stronger contributions from Ehrlich-pathway aldehydes and alcohols: 3-methylbutanal (~2.0 × 10^3^) imparting malty–fermented nuances, 2-methylbutanal and 3-methyl-1-butanol contributing nutty/fermented tones, and benzaldehyde (~1.1 × 10^2^) adding almond-nutty notes. Linalool (~3.8 × 10^2^) contributed a light floral–citrus component. Together, these reflect the catabolism of branched-chain and aromatic amino acids (leucine, isoleucine, phenylalanine) *via* the Ehrlich pathway, which proceeds readily in actively growing fungal mycelia. This pattern is consistent with the higher protein content of Bambara groundnuts, which provides amino-acid precursors for aldehyde and higher-alcohol formation *via* the Ehrlich pathway [8]. These substrate-dependent shifts in volatile formation are consistent with observations in other basidiomycetes, where mycelia similarly show distinct metabolic and flavour-active responses when grown on different plant substrates [12,13].

When considering temporal trends in volatile production, several analytes exhibited fluctuations over the cultivation period. These patterns should be interpreted cautiously, given the semi-quantitative nature of HS-SPME and the variability highlighted by the standard deviations reported in Appendix A. For example, compounds such as 3-octanol peaked at day 12 in both mycelial matrices (to ~31.0–37.5 µg/g), while several high-impact aldehydes (e.g., (E)-2-octenal, benzaldehyde) and alcohols (1-hexanol, 3-methyl-1-butanol) reached maxima around days 10–14, depending on the substrate. The data suggest that certain volatiles could undergo mid-growth intensification, which is plausible given the developmental and enzymatic shifts in *Pleurotus* C8 biosynthesis (e.g., stage-linked patterns involving lipoxygenase and oxylipins) reported for fruiting bodies and mycelia [7,40], but the patterns remain indicative rather than definitive. Confirmation would require externally calibrated quantitation or dynamic flux analysis.

No sulphur-containing volatiles (e.g., dimethyl disulfide, methional) were detected in the fresh samples, consistent with reports that thermal processing generates Maillard-derived pyrazines, Strecker aldehydes and sulphur compounds in oyster mushrooms [6,37,41]. Future work incorporating controlled heating could assess how substrate-driven precursor differences translate into heat-generated flavour notes in mycelia.

Overall, 1-octen-3-one, 3-methylbutanal, 3-octanone, 2,4-decadienal, 1-octen-3-ol and 3-octanol appear to drive the sensory identity across matrices, while substrate modulates pathway emphasis: amaranth accentuated oxylipin-linked, mushroom/green/fatty C8 intensity, while Bambara accentuated amino acid-derived Ehrlich aldehydes and higher alcohols, leading to malty, nutty and lightly floral profiles. Compared with the fruiting body’s classic fresh mushroom/green character, amaranth mycelia expressed a more intense mushroom–ketonic profile. In contrast, Bambara mycelia expressed a warmer nutty/fermented profile with reduced C8 dominance. These insights demonstrate how media composition can be used to steer mycelial flavour development; however, quantitative validation and sensory evaluation remain essential next steps.

### 3.3. Protein Content

**Figure 2:**
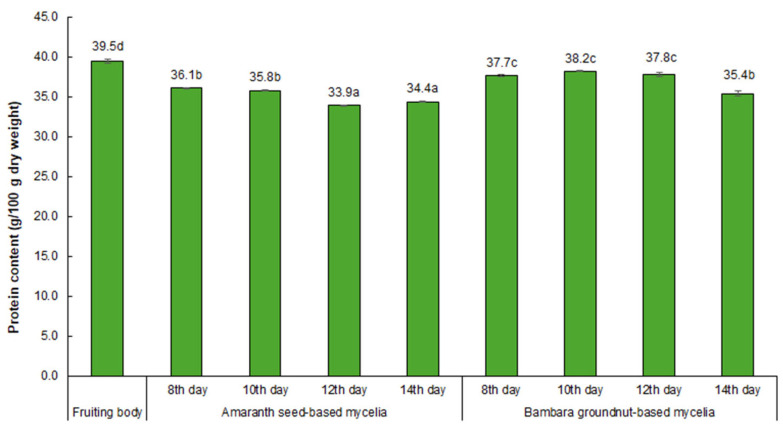
Protein content (g/100 g dry weight) in *P*. *ostreatus* fruiting body and mycelia cultivated on amaranth seed-based or Bambara groundnut-based media over four culture periods. Numbers on bars represent means of three replicates and different letters indicate significant differences (*p* ≤ 0.05).

The results of the fruiting body and mycelial protein contents are presented in Figure 2. The fruiting body exhibited a higher protein content (39.5%) than the mycelial samples. This disparity may be attributed to differences in nutrient availability between solid-state fruiting body substrates and liquid mycelial culture media, which influence protein accumulation. The wheat straw substrate used for fruiting body cultivation was supplemented with 15% wheat bran, a method shown to enhance the protein content of *P. ostreatus* fruiting bodies [42].

The mycelial protein content is influenced by both the culture medium and the cultivation period. Mycelia cultivated on Bambara groundnut-based media produced the highest protein content (up to 38.2%) between days 8 and 12. In contrast, mycelia grown on amaranth seed-based media yielded slightly lower values (36.1% and 35.8% on days 8 and 10, respectively), with no significant difference between these two time points (*p* > 0.05), suggesting that this medium maintains relatively consistent protein synthesis early in the culture period [43]. In mycelia cultured on Bambara groundnut-based media, the persistently elevated protein concentrations observed up to day 12 suggest that this substrate facilitates prolonged protein biosynthetic activity across an extended cultivation period. Differences in water-soluble nitrogen-containing compounds between amaranth- and Bambara groundnut-based media could contribute to the observed protein differences and should be investigated in future studies.

The decline in protein content observed on days 12 and 14 across both media likely reflects a progressive reduction in the carbon-to-nitrogen ratio as nutrient reserves become exhausted, representing a metabolic imbalance, which is a condition known to trigger autolysis and internal protein catabolism in filamentous fungi [44,45]. Additional factors, including microaerophilic or anaerobic zones within dense mycelial mats, have also been associated with autolytic responses [46]. Furthermore, extended cultivation may expose mycelia to physiological stress associated with the lag phase or transition to the stationary phase, which, although not extensively characterised in *P. ostreatus*, has been linked to metabolic slowdown and stress signalling in other fungi [47].

### 3.4. Amino Acid Composition

The amino acid composition of the fruiting body and mycelia cultivated on amaranth seed- or Bambara groundnut-based media (g/100 g protein, dry-weight basis) is presented in Table 4. In the fruiting body, leucine + isoleucine (6.56 g/100 g) and lysine (4.05 g/100 g) were the dominant indispensable amino acids, while methionine (0.51 g/100 g) was the lowest. This pattern resembles published *P. ostreatus* fruiting-body data, in which leucine is typically the most abundant essential amino acid and sulphur amino acids occur at comparatively low levels [48].

A similar qualitative hierarchy of amino acids was observed in the mycelial samples, irrespective of substrate. Amaranth-grown mycelia contained 9.09–10.25 g/100 g leucine + isoleucine and 4.46–5.75 g/100 g lysine, while Bambara-grown mycelia contained 8.66–8.99 g/100 g leucine + isoleucine and 4.93–5.47 g/100 g lysine. These values are comparable to those reported for *P. ostreatus* cultivated in submerged liquid culture using an amaranth flour medium, where leucine + isoleucine reached 8.5 g/100 g and lysine approximately 5.2 g/100 g [49].

With respect to dispensable amino acids, the profiles of the fruiting body and mycelia followed characteristic *P. ostreatus* patterns, with glutamic acid and aspartic acid being dominant. The fruiting body contained 8.68 g/100 g glutamic acid and 5.54 g/100 g aspartic acid, whereas amaranth-grown mycelia contained 13.99–17.96 g/100 g glutamic acid and 7.20–8.11 g/100 g aspartic acid. Mycelia grown on Bambara groundnut-based medium exhibited similarly high levels of glutamic acid (9.42–12.74 g/100 g) and aspartic acid (7.59–7.97 g/100 g). This is in accordance with submerged culture observations, where these two amino acids were also the most abundant in *P. ostreatus* mycelia grown in an amaranth flour-based medium [49] and in peat extract or synthetic media [50].

To assess protein quality, the ratio of indispensable to dispensable amino acids (IAA:DAA) was calculated. The fruiting body exhibited a ratio of 0.68, whereas amaranth-grown mycelia ranged from 0.60 to 0.71 (mean = 0.65), and Bambara-grown mycelia from 0.68 to 0.77 (mean = 0.71). These values indicate that mycelial biomass produced under the tested liquid-state conditions displays a balance between indispensable and dispensable amino acids comparable to that of the fruiting body. Reported IAA:DAA ratios for *P. ostreatus* vary widely in the literature. In fruiting bodies, very low indispensable-to-dispensable ratios (≈0.11) have been calculated from datasets in which non-essential amino acids strongly predominate [51]. In contrast, amino acid compositions reported for submerged *P. ostreatus* mycelia cultivated on amaranth-based media [49] yield calculated IAA:DAA ratios well above unity (≈2.0–2.5), reflecting relative enrichment of indispensable amino acids. More recent species-screening studies of *Pleurotus* fruiting bodies [52,53] similarly indicate dominance of dispensable amino acids, with calculated IAA:DAA ratios generally below 1.0 across multiple species. Collectively, these comparisons suggest that the broad range of reported IAA:DAA values could reflect differences in biological matrix as well as analytical challenges associated with amino acid determination. Nevertheless, the current results suggest that the IAA:DAA in mycelia is similar to that in the fruiting body under the conditions of the experiment reported in this paper.

**Figure 3:**
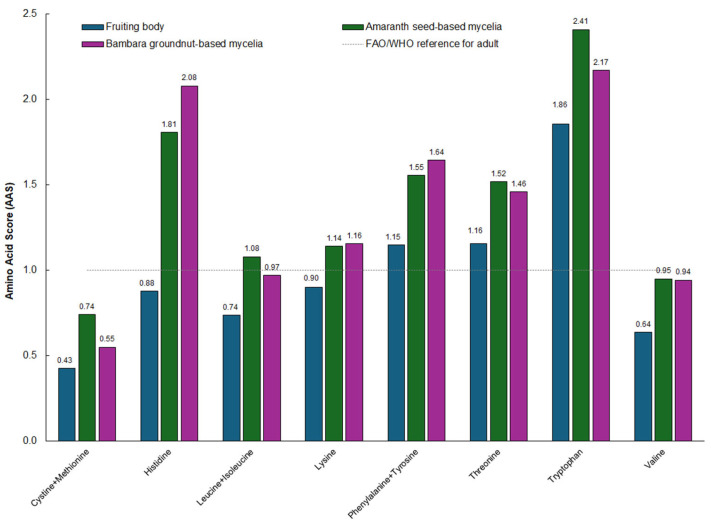
Amino acid scores (AASs) of *P*. *ostreatus* fruiting bodies and mycelia cultivated on amaranth seed-based or Bambara groundnut-based media.

Amino acid scores (AASs) provided further insight into the nutritional quality of the proteins (Figure 3). Because AAS values greater than 1 exceed the human reference requirement, only those <1 were used to identify limiting amino acids.

In all matrices, cystine + methionine produced the lowest AAS values (fruiting body: 0.43; amaranth mycelia: 0.74; Bambara mycelia: 0.55), confirming sulphur amino acids as the primary limiting factor in *P. ostreatus*. Histidine, leucine + isoleucine, lysine, threonine and valine all displayed AAS values below 1 in the fruiting body, while AASs were consistently higher in the mycelia than in the fruiting body. This pattern is consistent with reports that basidiomycete mycelial biomass typically exhibits improved indispensable amino acid representation relative to fruiting bodies, a trend supported by protein digestibility–corrected amino acid score (PDCAAS) assessments in other *Pleurotus* species, such as *P. pulmonarius* [13]. Collectively, the IAA:DAA ratios and AAS profiles indicate that both amaranth- and Bambara-grown mycelia provide a strong representation of most indispensable amino acids and a consistent limitation only in sulphur amino acids.

**Figure 4:**
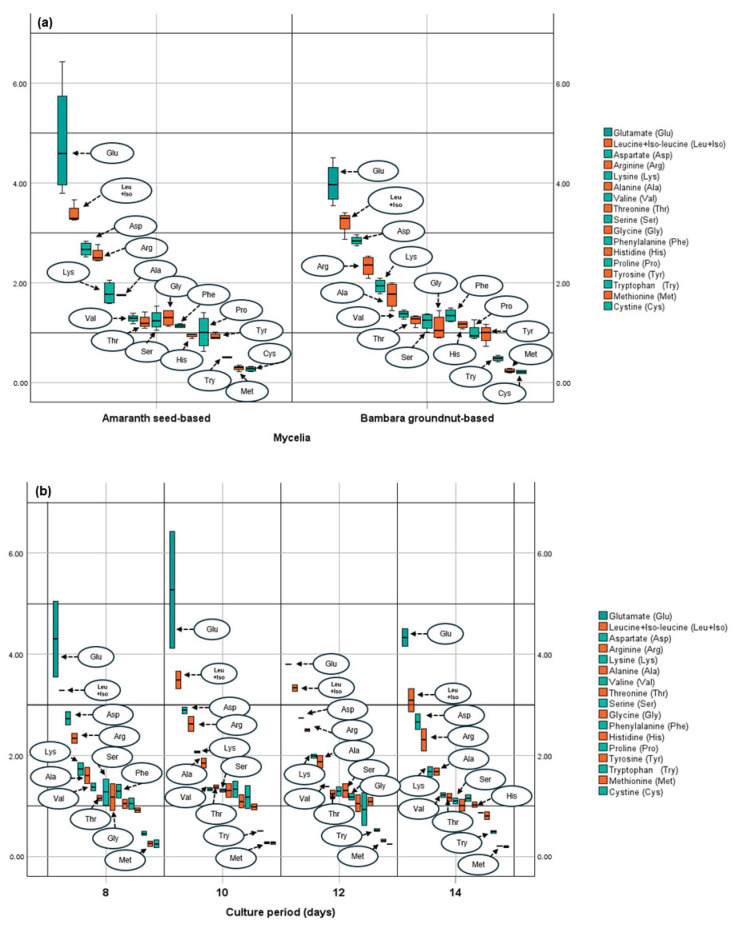
Box plots of the 17 amino acids in *P. ostreatus* mycelia (g/100 g mycelia, dry weight basis) as influenced by (**a**) culture on amaranth seed-based or Bambara groundnut-based media and (**b**) culture period (days 8, 10, 12, and 14). Each box plot illustrates the distribution of values for each concentration, highlighting key quantiles. The lower and upper edges of the box correspond to the 25th (first quantile) and 75th (third quantile) percentiles, respectively. The line within the box marks the median (second quantile) composition. The uppermost and lowermost lines (whiskers) extend to values within 1.5 times the interquartile range above the 75th percentile and below the 25th percentile.

Figure 4 provides an overview of the distribution of amino acids within the mycelial samples, illustrating the effects of culture medium (Figure 4a) and cultivation time (Figure 4b).

Amaranth seed-based media produced wider box plots and longer whiskers for several amino acids, indicating greater spread or variability in concentrations across the culture period, whereas Bambara-based media showed narrower distributions, suggesting more uniform amino acid levels. When data are grouped by day, glutamic acid peaked on day 10; however, the box plots provide a descriptive overview of dispersion rather than evidence of systematic changes over time.

Beyond nutritional value, several amino acids contribute directly to flavour-active volatile formation. Branched-chain amino acids (leucine, isoleucine, valine) are precursors to aroma-active aldehydes and alcohols formed through amino-acid catabolism in basidiomycetes, including 3-methylbutanal, 3-methyl-1-butanol, 2-methylbutanal and 2-methyl-1-butanol [6,8], all of which were detected in this study (Section 3.2). Phenylalanine is converted into benzaldehyde [6], which was also detected. Conversely, the low concentrations of sulphur amino acids (methionine, cystine) correspond with the absence of sulphur-containing volatiles in the profiles of fresh samples. The consistently high proportions of glutamic acid and aspartic acid, the major contributors to umami taste in *Pleurotus* species [54], suggest that *P. ostreatus* mycelia may function as natural umami-enhancing ingredients. Their strong representation complements the aroma-active volatile compounds identified in both fruiting body and mycelial samples, highlighting the potential of liquid-state mycelial biomass as a nutritionally balanced and flavour-relevant ingredient.

## 4. Conclusions

This study demonstrates that cultivating *P. ostreatus* mycelia on amaranth seed- and Bambara groundnut-based liquid media as surface cultures offers a nutritionally robust and flavour-active biomass. Mycelia generated a broader volatile profile than the fruiting body, including 35 additional compounds, highlighting the ability of liquid-state cultivation and substrate composition to shape fungal secondary metabolism. Clear substrate effects were evident: amaranth-based media supported a more intense mushroom-like, green and fatty C8 profile associated with oxylipin pathways, whereas Bambara groundnut-based media favoured aldehydes and higher alcohols characteristic of Ehrlich-type amino-acid catabolism, resulting in malty, nutty and lightly floral notes.

The amino-acid composition of the mycelia was qualitatively similar to that of the fruiting body, with leucine + isoleucine and lysine dominating the indispensable fraction, and glutamic acid and aspartic acid remaining the most abundant dispensable amino acids. The IAA:DAA ratios of between 0.60 and 0.77 indicated that dispensable amino acids were present in higher proportions than indispensable amino acids. The amino acid scores confirmed sulphur-containing amino acids (cystine + methionine) as the limiting components in mycelia, while histidine, lysine, tryptophan, threonine and phenylalanine + tyrosine met or exceeded reference requirements. The high levels of glutamate and aspartate underscore the potential of the mycelial biomass not only as a protein contributor but also as a natural umami-enhancing ingredient in food formulations.

Together, these findings highlight that surface liquid-state mycelial cultivation on plant-based media derived from these underutilised crops enables the production of *P. ostreatus* biomass with improved amino acid profiles, distinct aroma signatures, and favourable levels of flavour-active precursors. The combined nutritional and volatile attributes of the mycelia, along with the rapid and substrate-flexible nature of liquid-state production, underscore the agricultural and economical relevance of *P. ostreatus* mycelial biomass as a value-added ingredient for flavour modulation, nutritional enhancement, and incorporation into emerging alternative protein and hybrid food systems.

## Figures and Tables

**Table 1:** Summary of mycelial biomass yield and protein content of *Pleurotus ostreatus* cultivated on selected plant-based media under liquid-state conditions, adapted from [14].

Growth Medium (Nutrient Source)	Peak Mycelial Yield (% Dry Weight Basis)	Peak Protein Content (g/100 g Dry Weight)
Amaranth seed flour	20–21	30–31
Bambara groundnut flour	19–21	29–30
Hemp seed flour	≤9	39–42
Flaxseed flour	≤16	~36
Jowar (sorghum) flour	≤13	~25

**Table 2:** Representative nutritional composition of amaranth seed and Bambara groundnut (dry weight basis).

Component	Amaranth Seed (*Amaranthus* spp.)	Bambara groundnut ^a^ (*Vigna subterranea*)	Key References
Protein (g/100 g)	12.3–15.9	18.6–21.9	[15,16,17,18]
Lipid (g/100 g)	5.8–7.1	7.1–7.8	[16,17,19]
Carbohydrates (g/100 g)	62.0–70.0	55.6–60.8	[15,18,19]
Dominant indispensable amino acids	Lysine-rich, methionine limiting	Legume-typical, lysine adequate, sulphur amino acids limiting	[18,20,21]
Major fatty acids (FAs)	Linoleic acid (C18:2): ~40–55% Oleic acid (C18:1): ~20–32% Total unsaturated FAs > 75%	Linoleic acid (C18:2): ~34–45% Oleic acid (C18:1): ~17–25% Palmitic acid (C16:0): ~20–25% Unsaturated FAs ~55–65%	[16,17]

^a^ Dehulled cotyledon.

**Table 3:** Aroma attributes and linear retention indices (RIs) of volatile compounds identified by gas chromatography–mass spectrometry (GC-MS) in *P. ostreatus* fruiting bodies and mycelia cultivated in nutrient media (amaranth seed-based or Bambara groundnut-based).

No. ^a^	Compound	RI_cal_. ^b^	RI_lib_. ^c^	Odour Description ^d^	Presence (√) or Absence (-) of Compound		
					Fruiting Body	Amaranth Seed-Based Mycelia	Bambara Groundnut-Based Mycelia
1	2-methylbutanal	927	917	Musty, chocolate, nutty, furfural and isovaleraldehyde-like with malty and fermented nuances	-	-	√
2	3-methylbutanal	933	921	Ethereal, aldehydic, chocolate, peach, fatty	√	√	√
3	3-methyl-2-butanone	942	938	Sharp solvent-like with green, herbal, fruity and dairy nuances	-	√	-
4	Ethanol	946	994	Alcoholic, ethereal	√	√	√
5	Pentanal	990	977	Diffusive, fermented, bready, fruity with berry nuances	-	√	-
6	Toluene	1058	1048	Sweet	√	√	√
7	2-methyldecane	1067	1057	N/A	-	√	√
8	3-methyldecane	1080	1070	N/A	-	√	√
9	Hexanal Undecane	1096	1087 1100	Green, fatty, leafy, vegetative, fruity, woody nuance (hexanal) Herbal, woody (undecane)	-	√	√
10	2-methyl-1-propanol	1114	1101	Ethereal, musty, phenolic	-	√	√
11	Methyl 4-methylpentanoate	1154	1136	Fruity, sweet, banana, pineapple, with cheese-like nuances	√	-	-
12	3-heptanone	1164	1167	Fruity, ethereal, ketonic, sweet with a musty cheese-like note	-	√	-
13	2-heptanone	1194	1178	Cheese, fruity, ketonic, green banana, with creamy nuance	-	√	√
14	Heptanal	1196	1189	Fresh, green, fruity	-	√	√
15	3-methyl-1-butanol	1215	1214	Fruity, fermented, winey	√	√	√
16	2-pentyl-furan	1237	1233	Fruity, green, earthy, beany with vegetable-like nuances	√	√	√
17	(E)-2-nonenal	1250	1545	Fatty, green, waxy, and vegetative with cucumber-melon, cereal notes, and a chicken fat nuance	-	√	√
18	1-pentanol	1254	1253	Pungent, fermented, bready, yeasty, fusel, winey and solvent-like	-	√	√
19	3-octanone	1266	1259	Musty, mushroom, ketonic, mouldy and cheesy fermented with a green, vegetative nuance	√	√	√
20	2-octanone	1293	1292	Musty, ketonic, bleu and parmesan cheese-like with woody, earthy and dairy nuances	-	√	-
21	Octanal	1298	1295	Aldehydic, waxy, citrus orange with a green peel-like nuance	-	√	√
22	4-methyl-6-hepten-3-one	1306	N/A	N/A	-	√	-
23	1-octen-3-one	1310	1308	Earthy, metallic, mushroom-like with vegetative nuances of cabbage and broccoli	√	√	-
24	5-methyl-5-hepten-3-one	1330	N/A	N/A	-	√	-
25	(Z)-2-heptenal	1331	1332	Green, fatty	√	√	√
26	6-methyl-5-hepten-2-one	1345	1341	Fruity, apple, musty, ketonic and creamy with slight cheesy and banana nuances	-	√	-
27	2-nonanone	1349	1366	Fresh, sweet, green, weedy, waxy, soapy	-	√	-
28	1-hexanol	1355	1355	Pungent, ethereal, fruity and alcoholic, sweet with a green top note	√	√	√
29	3-octanol	1394	1396	Earthy, mushroom, dairy, musty, creamy, waxy, with a slight fermented green minty nuance	√	√	√
30	Nonanal	1402	1401	Waxy, aldehydic, citrus with a fresh, slightly green lemon peel-like nuance, and a cucumber fattiness	-	√	√
31	4-ethylcyclohexanone	1406	N/A	N/A	-	√	-
32	Methyl-1-methlylcyclopropyl ketone	1413	N/A	N/A	-	√	-
33	4-methyl-6-hepten-3-ol	1415	N/A	N/A	-	√	√
34	(E)-2-octenal	1434	1437	Fatty, green, herbal	√	√	√
35	1-octen-3-ol	1449	1453	Earthy, green, oily, vegetative and fungal/mushroom-like	√	√	√
36	1-heptanol	1455	1460	Musty, pungent, leafy green, with vegetative and fruity nuances	-	√	√
37	2-ethyl-1-hexanol	1490	1494	Fresh, citrus, floral, oily, sweet	-	√	-
38	Benzaldehyde	1538	1533	Almond, fruity, powdery, and nutty	√	√	√
39	Linalool	1546	1537	Fresh, floral-woody, sweet, citrus	-	√	√
40	1-octanol	1557	1562	Waxy, green, citrus, aldehydic and floral with sweet, fatty, coconut nuance	√	√	√
41	2-undecanone	1604	1605	Waxy, fruity, floral, creamy, ketonic with fatty pineapple nuances	-	√	-
42	(Z)-2-octen-1-ol*	1615	1552	Sweet, floral	-	-	√
43	(E)-2-octen-1-ol	1617	1621	Green, vegetative	√	√	√
44	Methyl benzoate	1632	1638	Aromatic, sweet, floral with a fruity undertone	-	√	√
45	Acetophenone	1663	1664	Sweet, almond, orange, cherry pit, honeysuckle, strawberry, vanilla nuance	-	-	√
46	Oxime-methoxy-phenyl	1759	N/A	N/A	-	√	√
47	2,4 decadienal	1816	1811	Fatty, citrus, nutty	-	√	√
48	Benzyl alcohol	1883	1877	Sweet, floral, fruity	-	√	√
49	Phenylethyl alcohol	1917	1916	Sweet, floral, fresh	-	√	√
50	Octanoic acid	2072	2068	Fatty, waxy, rancid, oily, vegetative, cheesy	-	√	-
51	Nonanoic acid	2173	2174	Cheesy, waxy, fatty	-	√	-
52	Methyl tridecanoate *	2210	1921	N/A	√	-	-

^a^ No: Numbers of compounds observed in GC-MS profiles. ^b^ RI_cal_.: Linear retention index as calculated using C_8_–C_40_ alkane mix range. ^c^ RI_lib_.: Linear retention index obtained from NIST library, ≥70% similarity * or literature (for asterisked compounds, RI not a perfect match). Abbreviation: N/A—Not available (retention index) from the NIST library. ^d^ Odour description obtained from [6,8,28]. Abbreviation: N/A—Not available (odour description) from peer-reviewed publications.

**Table 4:** Amino acid composition (g/100 g total protein, dry weight basis) of *P. ostreatus* fruiting bodies and mycelia cultivated on amaranth seed-based or Bambara groundnut-based media over the four culture periods.

Amino Acids	Fruiting Body	Amaranth Seed-Based Mycelia					Bambara Groundnut-Based Mycelia				
		8th Day	10th Day	12th Day	14th Day	Mean ± SD	8th Day	10th Day	12th Day	14th Day	Mean ± SD
**Indispensable**											
Histidine	1.32	2.66	2.71	2.63	2.85	2.71 ± 0.10	3.02	3.19	3.23	3.05	3.12 ± 0.10
Leucine + Isoleucine	6.56	9.09	10.25	9.62	9.62	9.64 ± 0.48	8.70	8.66	8.99	8.11	8.62 ± 0.37
Lysine	4.05	4.46	5.73	5.75	4.62	5.14 ± 0.70	4.93	5.47	5.37	5.03	5.20 ± 0.26
Methionine	0.51	0.86	0.84	1.03	0.64	0.84 ± 0.16	0.56	0.68	0.77	0.56	0.64 ± 0.10
Phenylalanine	2.30	3.21	3.30	3.30	3.20	3.25 ± 0.06	3.79	3.90	3.31	3.45	3.61 ± 0.28
Threonine	2.66	3.35	3.97	3.48	3.17	3.49 ± 0.34	2.94	3.51	3.49	3.53	3.37 ± 0.28
Tryptophan	1.11	1.39	1.45	1.47	1.48	1.45 ± 0.04	1.11	1.31	1.46	1.33	1.30 ± 0.14
Valine	2.48	3.60	3.63	4.10	3.43	3.69 ± 0.29	3.87	3.59	3.68	3.59	3.68 ± 0.13
**Dispensable**											
Alanine	4.20	4.90	4.92	5.16	5.09	5.02 ± 0.13	3.85	5.10	5.29	4.55	4.70 ± 0.65
Arginine	3.80	6.76	7.74	7.32	7.35	7.29 ± 0.40	5.97	6.49	6.69	5.90	6.26 ± 0.30
Aspartic acid	5.54	7.20	7.91	8.11	7.33	7.64 ± 0.44	7.59	7.75	7.25	7.97	7.64 ± 0.30
Cystine	0.43	0.91	0.84	0.77	0.64	0.79 ± 0.12	0.48	0.65	0.63	0.51	0.57 ± 0.09
Glutamic acid	8.68	13.99	17.96	11.18	12.06	13.80 ± 3.01	9.42	10.79	10.08	12.74	10.76 ± 1.44
Glycine	2.41	3.99	4.05	3.48	3.28	3.70 ± 0.38	2.41	3.09	3.84	2.54	2.97 ± 0.65
Proline	1.14	3.21	3.94	1.83	2.50	2.87 ± 0.91	2.49	2.49	3.33	2.49	2.70 ± 0.42
Serine	2.51	4.27	3.58	3.54	3.05	3.61 ± 0.50	2.71	3.51	3.65	3.28	3.29 ± 0.42
Tyrosine	2.05	2.44	2.60	2.98	2.59	2.65 ± 0.23	2.57	2.75	3.10	2.06	2.62 ± 0.43
**IAA:DAA**	**0.68**	**0.60**	**0.60**	**0.71**	**0.66**	**0.65 ± 0.05**	**0.77**	**0.71**	**0.69**	**0.68**	**0.71 ± 0.04**

SD—Standard deviation; IAA:DAA—Ratio of indispensable amino acids to dispensable amino acids.

## Data Availability

The original contributions presented in this study are included in the article. Further inquiries can be directed to the corresponding author.

## Abbreviations

The following abbreviations are used in this manuscript:

AAS	Amino Acid Score
IAA	Indispensable Amino Acid
DAA	Dispensable Amino Acid
PDCAAS	Protein Digestibility–Corrected Amino Acid Score
GC–MS	Gas Chromatography–Mass Spectrometry
HS-SPME	Headspace Solid-Phase Microextraction
ToF-MS	Time-of-Flight Mass Spectrometry
RI	Retention Index
RRI	Relative Retention Index
m/z	Mass-to-Charge Ratio
OAV	Odour Activity Value
MEA	Malt Extract Agar
PDMS/DVB-OC	Polydimethylsiloxane/Divinylbenzene Overcoated
LOX	Lipoxygenase
HPOD	Hydroperoxy-Octadecadienoic Acid

## Appendix A

**Table A1:** Volatile compounds (µg/g fresh weight) in *P. ostreatus* fruiting bodies and mycelia cultivated on nutrient media (amaranth seed-based or Bambara groundnut-based) over four culture periods.

Class of Compound	Compound Name	Odour Thresholds ^a^	Fruiting Body ^b^	Amaranth Seed-Based Mycelia ^b^				Bambara Groundnut-Based Mycelia ^b^			
				8th Day	10th Day	12th Day	14th Day	8th Day	10th Day	12th Day	14th Day
Acids	Nonanoic acid	3.47	-	-	0.23 ± 0.19 ^AB,a^	0.30 ± 0.06 ^B,a^	0.14 ± 0.11 ^AB,a^	-	-	-	-
	Octanoic acid	1.83	-	-	-	0.11 ± 0.09 ^A,a^	-	-	-	-	-
Alcohols	1-hexanol	0.444	0.17 ± 0.08 ^A,a^	4.40 ± 1.61 ^A,bc^	6.78 ± 1.49 ^A,d^	5.58 ± 1.57 ^A,b^	6.91 ± 2.11 ^A,d^	3.46 ± 2.88 ^A,a^	5.59 ± 0.69 ^A,ab^	4.04 ± 0.21 ^A,ab^	7.77 ± 0.66 ^A,abc^
	1-heptanol	0.342	-	0.11 ± 0.10 ^A,a^	0.22 ± 0.09 ^A,a^	0.20 ± 0.09 ^A,a^	0.25 ± 0.85 ^A,a^	0.18 ± 0.15 ^A,a^	0.30 ± 0.07 ^A,a^	0.20 ± 0.04 ^A,a^	0.34 ± 0.14 ^A,a^
	1-octanol	0.149	0.16 ± 0.04 ^A,a^	0.94 ± 0.28 ^ABC,abc^	1.47 ± 0.49 ^BC,abc^	1.09 ± 0.32 ^ABC,a^	1.64 ± 0.21 ^C,abc^	0.45 ± 0.37 ^AB,a^	0.55 ± 0.03 ^AB,a^	0.80 ± 0.19 ^ABC,a^	0.76 ± 0.17 ^ABC,a^
	1-pentanol	0.514	-	-	0.47 ± 0.41 ^AB,ab^	0.44 ± 0.11 ^AB,a^	0.52 ± 0.14 ^AB,a^	0.57 ± 0.49 ^AB,a^	0.63 ± 0.19 ^AB,a^	0.35 ± 0.18 ^AB,a^	1.13 ± 0.61 ^B,a^
	3-octanol	0.0705	10.3 ± 2.98 ^AB,b^	23.3 ± 2.73 ^AB,c^	24.0 ± 4.62 ^AB,e^	31.0 ± 6.96 ^AB,c^	26.6 ± 1.55 ^AB,e^	7.42 ± 4.84 ^A,a^	18.8 ± 10.9 ^AB,c^	37.5 ± 6.15 ^B,d^	33.2 ± 6.95 ^AB,bcd^
	2-ethyl-1-hexanol	0.16	-	-	0.02 ± 0.01 ^A,a^	0.03 ± 0.01 ^A,a^	-	-	-	-	-
	1-octen-3-ol	0.00274	1.33 ± 0.63 ^AB,a^	1.63 ± 0.29 ^AB,abc^	2.26 ± 0.53 ^B,abc^	1.30 ± 0.53 ^AB,a^	1.15 ± 0.18 ^AB,ab^	0.75 ± 0.57 ^A,a^	0.40 ± 0.09 ^A,a^	0.39 ± 0.11 ^A,a^	0.71 ± 0.09 ^A,a^
	2-methyl-1-propanol	0.346	-	0.09 ± 0.09 ^A,a^	0.27 ± 0.09 ^AB,a^	0.33 ± 0.05 ^B,a^	0.27 ± 0.02 ^AB,a^	0.26 ± 0.12 ^AB,a^	0.20 ± 0.01 ^AB,a^	0.19 ± 0.06 ^AB,a^	0.33 ± 0.15 ^AB,a^
	3-methyl-1-butanol	0.466	0.20 ± 0.17 ^A,a^	1.37 ± 0.55 ^A,abc^	2.09 ± 0.55 ^A,abc^	2.57 ± 1.08 ^A,ab^	3.24 ± 0.47 ^A,c^	3.09 ± 1.08 ^A,a^	7.89 ± 1.26 ^A,b^	6.43 ± 2.36 ^A,b^	9.81 ± 3.79 ^A,abc^
	4-methyl-6-hepten-3-ol		-	0.08 ± 0.03 ^A,a^	0.11 ± 0.04 ^A,a^	0.11 ± 0.02 ^A,a^	0.11 ± 0.03 ^A,a^	0.10 ± 0.08 ^A,a^	0.10 ± 0.04 ^A,a^	0.30 ± 0.10 ^B,a^	-
	(E)-2-octen-1-ol	0.018	0.53 ± 0.13 ^AB,a^	1.24 ± 0.05 ^BC,abc^	1.71 ± 0.74 ^C,abc^	0.87 ± 0.29 ^ABC,a^	1.54 ± 0.29 ^C,abc^	0.28 ± 0.19 ^AB,a^	-	0.50 ± 0.33 ^AB,a^	0.37 ± 0.35 ^AB,a^
	(Z)-2-octen-1-ol	0.00866	-	-	-	-	-	0.02 ± 0.01 ^A,a^	0.22 ± 0.12 ^B,a^	0.02 ± 0.01 ^A,a^	-
	Ethanol	21.9	0.02 ± 0.01 ^A,a^	1.98 ± 0.60 ^C,abc^	0.81 ± 0.06 ^AB,ab^	0.98 ± 0.11 ^B,a^	0.57 ± 0.08 ^AB,a^	0.62 ± 0.28 ^AB,a^	0.74 ± 0.04 ^AB,a^	0.82 ± 0.04 ^AB,a^	0.77 ± 0.56 ^AB,a^
	Phenylethyl alcohol	2.6	-	-	0.02 ± 0.02 ^A,a^	-	0.04 ± 0.04 ^A,a^	0.09 ± 0.04 ^A,a^	0.34 ± 0.13 ^A,a^	-	0.41 ± 0.23 ^A,a^
Aldehydes	2-methylbutanal	0.00154	-	-	-	-	-	0.04 ± 0.03 ^A,a^	0.01 ± 0.00 ^A,a^	-	0.07 ± 0.07 ^A,a^
	3-methylbutanal	0.00153	0.20 ± 0.10 ^A,a^	0.30 ± 0.05 ^A,a^	0.66 ± 0.44 ^A,ab^	0.34 ± 0.18 ^A,a^	0.41 ± 0.20 ^A,a^	2.17 ± 2.24 ^A,a^	1.79 ± 0.21 ^A,ab^	1.08 ± 0.40 ^A,a^	2.99 ± 1.08 ^A,ab^
	(E)-2-nonenal	0.000185	-	-	0.09 ± 0.02 ^D,a^	0.08 ± 0.04 ^CD,a^	0.06 ± 0.01 ^BCD,a^	0.03 ± 0.02 ^ABC,a^	0.03 ± 0.01 ^ABC,a^	0.02 ± 0.01 ^AB,a^	-
	2,4-decadienal	0.0000955		-	0.01 ± 0.00 ^A,a^	0.01 ± 0.01 ^A,a^	0.07 ± 0.08 ^A,a^	0.12 ± 0.10 ^A,a^	-	-	-
	Benzaldehyde	0.404	0.06 ± 0.03 ^A,a^	4.49 ± 2.51 ^A,c^	5.73 ± 4.45 ^A,cd^	1.94 ± 0.94 ^A,ab^	2.71 ± 1.48 ^A,bc^	32.2 ± 12.1 ^A,b^	17.1 ± 5.05 ^A,c^	23.2 ± 3.13 ^A,c^	43.4 ± 13.1 ^A,d^
	Heptanal	0.00585	-	-	0.19 ± 0.15 ^A,a^	0.12 ± 0.04 ^A,a^	0.15 ± 0.08 ^A,a^	0.18 ± 0.18 ^A,a^	0.09 ± 0.03 ^A,a^	0.07 ± 0.04 ^A,a^	-
	Nonanal	0.00279		-	0.13 ± 0.11 ^A,a^	0.05 ± 0.01 ^A,a^	0.04 ± 0.02 ^A,a^	0.18 ± 0.27 ^A,a^	0.09 ± 0.06 ^A,a^	-	-
	Octanal	0.00229	-	-	0.12 ± 0.04 ^AB,a^	0.11 ± 0.03 ^AB,a^	0.12 ± 0.01 ^AB,a^	0.07 ± 0.01 ^AB,a^	0.08 ± 0.05 ^AB,a^	0.08 ± 0.01 ^AB,a^	-
	Pentanal	0.011	-	-	0.13 ± 0.09 ^B,a^	0.03 ± 0.03 ^AB,a^	0.04 ± 0.04 ^AB,a^	-	-	-	-
	(E)-2-octenal	0.00315	0.21 ± 0.05 ^A,a^	0.32 ± 0.14 ^A,a^	0.96 ± 0.78 ^A,ab^	0.47 ± 0.21 ^A,a^	0.93 ± 0.46 ^A,a^	1.30 ± 2.02 ^A,a^	1.05 ± 1.03 ^A,a^	0.73 ± 0.19 ^A,a^	1.05 ± 1.00 ^A,a^
	(Z)-2-heptenal	0.000775	0.07 ± 0.02 ^A,a^	0.10 ± 0.00 ^A,a^	0.41 ± 0.23 ^A,ab^	0.23 ± 0.15 ^A,a^	0.34 ± 0.15 ^A,a^	0.41 ± 0.59 ^A,a^	0.17 ± 0.08 ^A,a^	0.09 ± 0.01 ^A,a^	-
Alkanes	2-methyldecane		-	-	0.02 ± 0.01 ^AB,a^	0.01 ± 0.01 ^A,a^	0.01 ± 0.01 ^A,a^	0.01 ± 0.01 ^A,a^	0.05 ± 0.02 ^AB,a^	0.02 ± 0.01 ^AB,a^	-
	3-methyldecane		-	-	-	0.09 ± 0.09 ^A,a^	0.01 ± 0.01 ^A,a^	0.02 ± 0.01 ^A,a^	0.02 ± 0.01 ^A,a^	0.01 ± 0.01 ^A,a^	-
Benzene	Oxime-methoxy-phenyl		-	1.06 ± 0.32 ^B,abc^	-	-	-	0.15 ± 0.18 ^A,a^	-	-	-
Esters	Methyl benzoate	0.0471	-	0.42 ± 0.07 ^A,ab^	0.19 ± 0.14 ^A,a^	0.19 ± 0.05 ^A,a^	0.23 ± 0.04 ^A,a^	1.12 ± 1.00 ^A,a^	0.49 ± 0.08 ^A,a^	0.59 ± 0.18 ^A,a^	1.22 ± 0.57 ^A,a^
	Methyl tridecanoate		0.04 ± 0.01 ^A,a^	-	-	-	-	-	-	-	-
	Methyl 4-methylpentanoate	0.0118	0.05 ± 0.02 ^A,a^	-	-	-	-	-	-	-	-
Aromatic compounds	Benzyl alcohol	3.636	-	0.15 ± 0.01 ^A,a^	0.19 ± 0.10 ^A,a^	0.11 ± 0.16 ^A,a^	0.34 ± 0.17 ^A,a^	1.18 ± 0.46 ^A,a^	2.06 ± 0.72 ^A,ab^	1.98 ± 0.94 ^A,ab^	3.23 ± 1.72 ^A,ab^
	Toluene	0.098	0.64 ± 0.30 ^A,a^	0.69 ± 0.11 ^A,abc^	0.86 ± 0.18 ^A,ab^	0.98 ± 0.19 ^A,a^	1.32 ± 0.14 ^A,ab^	1.23 ± 1.16 ^A,a^	0.87 ± 0.07 ^A,a^	0.67 ± 0.13 ^A,a^	0.71 ± 0.13 ^A,a^
	2-pentyl-furan	0.00394	0.01 ± 0.01 ^A,a^	-	0.21 ± 0.16 ^A,a^	0.15 ± 0.08 ^A,a^	0.15 ± 0.10 ^A,a^	0.35 ± 0.40 ^A,a^	0.11 ± 0.05 ^A,a^	0.13 ± 0.02 ^A,a^	0.26 ± 0.13 ^A,a^
Ketones	2-heptanone	0.0329	-	-	0.02 ± 0.02 ^A,a^	0.02 ± 0.01 ^A,a^	0.01 ± 0.01 ^A,a^	0.03 ± 0.03 ^A,a^	0.02 ± 0.01 ^A,a^	-	-
	3-heptanone	0.0278	-	-	0.02 ± 0.01 ^A,a^	0.03 ± 0.01 ^A,a^	0.04 ± 0.02 ^A,a^	-	-	-	-
	2-octanone	0.0149	-	-	0.03 ± 0.04 ^AB,a^	0.04 ± 0.02 ^AB,a^	0.06 ± 0.03 ^B,a^	-	-	-	-
	3-octanone	0.0256	14.5 ± 5.28 ^A,c^	36.4 ± 7.49 ^A,d^	33.3 ± 5.72 ^A,f^	35.7 ± 4.60 ^A,d^	35.0 ± 1.91 ^A,f^	8.95 ± 3.21 ^A,a^	18.2 ± 6.94 ^A,c^	33.9 ± 6.61 ^A,d^	37.4 ± 6.23 ^A,cd^
	2-nonanone	0.0334	-	-	0.02 ± 0.01 ^A,a^	0.02 ± 0.01 ^A,a^	0.05 ± 0.01 ^A,a^	-	-	-	-
	1-octen-3-one	0.0000292	0.04 ± 0.17 ^A,a^	-	0.10 ± 0.02 ^B,a^	0.06 ± 0.01 ^A,a^	0.12 ± 0.03 ^B,a^	-	-	-	-
	2-undecanone	0.112	-	-	0.05 ± 0.03 ^A,a^	-	-	-	-	-	-
	4-ethylcyclohexanone		-	-	0.02 ± 0.01 ^A,a^	0.02 ± 0.01 ^A,a^	0.04 ± 0.02 ^B,a^	-	-	-	-
	3-methyl-2-butanone	0.798	-	-	0.02 ± 0.01 ^A,a^	0.02 ± 0.01 ^A,a^	0.02 ± 0.01 ^A,a^	-	-	-	-
	4-methyl-6-hepten-3-one		-	-	0.03 ± 0.01 ^A,a^	0.03 ± 0.01 ^A,a^	0.04 ± 0.02 ^A,a^	-	-	-	-
	5-methyl-5-hepten-3-one		-	-	0.04 ± 0.02 ^B,a^	0.02 ± 0.01 ^A,a^	0.02 ± 0.01 ^A,a^	-	-	-	-
	6-methyl-5-hepten-2-one	0.194	-	-	0.06 ± 0.03 ^B,a^	0.03 ± 0.01 ^AB,a^	0.04 ± 0.01 ^B,a^	-	-	-	-
	Methyl-1-methlylcyclopropyl ketone		-	-	-	0.07 ± 0.01 ^A,a^	0.14 ± 0.05 ^B,a^	-	-	-	-
	Acetophenone	0.0937	-	-	-	-	-	0.09 ± 0.14 ^A,a^	-	-	-
Terpene	Linalool	0.00369	-	0.44 ± 0.20 ^A,ab^	0.46 ± 0.46 ^A,ab^	0.63 ± 0.36 ^A,a^	0.32 ± 0.07 ^A,a^	1.37 ± 1.25 ^A,a^	1.41 ± 0.31 ^A,a^	0.94 ± 0.35 ^A,a^	0.92 ± 0.81 ^A,a^
Coelution	Hexanal	0.00568	-	2.24 ± 0.84 ^A,abc^	4.63 ± 2.35 ^A,bcd^	2.06 ± 0.50 ^A,ab^	2.74 ± 0.76 ^A,bc^	3.48 ± 1.28 ^A,a^	1.55 ± 0.82 ^A,ab^	1.09 ± 0.09 ^A,a^	3.05 ± 1.49 ^A,ab^
	Undecane	10									

Results are presented as mean ± SD of three replicates. Different superscript capital letters attached to the values indicate significant difference within the same row, and different superscript lower-case letters attached to the values indicate significant difference within columns. Significant difference is at *p* ≤ 0.05 according to Duncan’s multiple range test. Not detected (-). ^a^ Water odour threshold values of aroma-active compounds sourced from [55]. Where multiple literature values or ranges were reported, a single representative water odour threshold was calculated as the geometric mean. ^b^ Odour activity value (OAV) was calculated as $OAV=\frac{concentration\;(\mu g/g)}{odor\;threshold\;(\mu g/g)}$ [8]. Values with concentrations exceeding the odour thresholds (OAV > 1) are shaded as follows: green (1–9), yellow (10–99), orange (100–999), red (1000–9999).
